# Physics-Constrained Reconstructions of Sunspot Number from Millennial-Scale Annual Heliospheric Modulation Potential

**DOI:** 10.1007/s11207-026-02731-0

**Published:** 2026-09-18

**Authors:** Chitradeep Saha, Mathew Owens, Mike Lockwood, Luke Barnard, Nicolas Brehm, Silvia Dalla, Konstantin Herbst, Raimund Muscheler, Jian Wang

**Affiliations:** 1 Department of Meteorology, University of Reading, Earley Gate, PO Box 243, Reading, RG6 6ET UK; 2 Laboratory of Ion Beam Physics, ETHZ, Zurich, Switzerland; 3 Jeremiah Horrocks Institute, University of Lancashire, Preston, PR1 2HE UK; 4 Centre for Planetary Habitability (PHAB), Department of Geosciences, University of Oslo, Oslo, Norway; 5 Lund University, Box 117, SE-221 00 Lund, Sweden; 6 Centre for Isotope Research, University of Groningen, Nijenborgh 6, 9747AG Groningen, the Netherlands

**Keywords:** Solar cycle, Sunspots, Galactic cosmic rays, Open solar flux, Heliospheric modulation

## Abstract

Long-term solar magnetic variability governs the space climate conditions. These variations also modulate the galactic cosmic ray influx reaching Earth and subsequently regulate the production of cosmogenic isotopes in Earth’s atmosphere. By harnessing the multi-millennial records of the abundances of these radio-isotopes stored in natural terrestrial reservoirs, such as tree wood and ice sheets, it is possible to reconstruct past solar magnetic activity predating the telescopic era. However, existing regression-based reconstruction methods using isotope data from time series extracted from the reservoirs are often applied to conditions outside the valid parameter range. This can result in a number of problems, including negative sunspot numbers during low solar activity phases, which correspond to statistically permissible extrapolation artefacts but have no physical meaning. To address this, we developed a Monte-Carlo inversion framework consisting of a sequence of physics-based semi-empirical forward models, supplemented by an Approximate Bayesian Computation-based posterior selection, to infer the most plausible temporal evolution of solar magnetic parameters. Our approach produces physically consistent, uncertainty-quantified reconstructions of annually resolved parameters, including sunspot numbers. We apply the method to two modulation potential datasets, namely geomagnetic record from 1845 – 2020, and an annual ^14^C estimate over the period 971 – 1932. Our new reconstruction method is designed to yield strictly non-negative sunspot numbers and shows close agreement with direct observations within uncertainty during the telescopic era. These results offer valuable insights into long-term solar variability and provide improved constraints for long-term solar dynamo modelling. The annual-scale reconstructions are also directly usable for solar irradiance estimation, terrestrial climate modelling, and studies of long-term Sun-Earth coupling.

**Supplementary Information:**

The online version contains supplementary material available at https://doi.org/10.1007/s11207-026-02731-0.

## Introduction

One of the most prominent signatures of long-term solar magnetic variability is the ≈ 11-year solar cycle, manifested by the rhythmic waxing and waning of the number of dark patches – sunspots – where magnetic field emerges through the Sun’s photosphere (Hathaway 2015). Large-scale photospheric magnetic fields extend into the corona, eventually filling the heliosphere as the heliospheric magnetic field (HMF), and channel the Sun’s particulate output in the interplanetary space (Owens and Forsyth 2013). Thus, the HMF establishes a causal connection between solar magnetic activity and the near-Earth space-climate conditions (Nandy et al. 2023). Owing to stochastic forcing of turbulent plasmas in the Sun’s convection zone (Saha, Mukhopadhyay, and Nandy 2025), solar magnetic cycles exhibit a wide range of variation in amplitude and – to a lesser extent – duration, and govern the space climate dynamics at Earth over centennial to millennial, and even longer, timescales (Usoskin 2023; Pevtsov et al. 2023).

Knowledge of past space climate is important from various perspectives. It is an important first step to understand the past severe space weather events (Lockwood et al. 2018; Owens et al. 2021; Cliver et al. 2022), the influence of solar forcings on the terrestrial climate (Kopp 2025; Lockwood and Ball 2020; Chatzistergos 2024), and to extend our understanding of solar magnetism to other sun-like stars (Nandy et al. 2021; Vidotto 2021; Amazo-Gómez et al. 2023; Alvarado-Gómez et al. 2025). Sunspot numbers (hereafter, SN) serve as an important proxy for studying large-scale and long-term solar magnetic activity; however, the longest continuous record of telescopic observations of sunspots dates back only to the past four centuries (Clette et al. 2014; Arlt and Vaquero 2020) and contains significant uncertainties prior to 1750 (Chatzistergos et al. 2017). Therefore, direct sunspot records prove insufficient for studying millennial-scale solar magnetic variability. Cosmogenic isotopes – such as ^14^C and ^10^Be stored in natural and independently dateable terrestrial reservoirs – record imprints of solar magnetic activity over geological timescales (Muscheler et al. 2007; Heaton et al. 2021; Brehm et al. 2021, 2025; Wang et al. 2026). These serve as the only indirect proxies of the Sun’s magnetic history in the distant past that we know of.

There exists a causal relationship between the level of solar magnetic activity and the production rate of the cosmogenic isotopes. The entirety of the heliosphere, the protective plasma bubble around our solar system formed by the solar wind and the embedded HMF, is relentlessly bombarded by high-energy galactic cosmic rays (GCRs) from outside the solar system. Some GCRs overcome both the protective magnetic shielding of HMF and the Earth’s own magnetosphere and permeate the Earth’s atmosphere where they interact with the atmospheric atoms and molecules forming a cascade of secondary cosmic ray particles, mainly high-energy neutrons and protons (Kovaltsov, Mishev, and Usoskin 2012; Herbst, Muscheler, and Heber 2017). These secondary particles collide with atmospheric nitrogen, oxygen and argon atoms to produce radionuclides through spallation and neutron-capture. These eventually find their way into various natural reservoirs: carbon dioxide formed with the ^14^C isotope is absorbed by trees as part of photosynthesis and ^10^Be, and ^36^Cl become attached to aerosols and precipitate into ice sheets and sediments. The intensity of GCRs incident on the Earth’s magnetosphere depends not only on the strength of the HMF but also the HMF tilt angle (Alanko-Huotari et al. 2007; Herbst, Muscheler, and Heber 2017; Owens et al. 2024b). A magnetically more active Sun creates a more effective HMF shielding against the GCR influx (Heber, Fichtner, and Scherer 2006; Potgieter 2013), as quantified by a semi-empirical parameter – heliospheric modulation potential (hereafter, ϕ) – arising from the force-field approximation (Gleeson and Axford 1968; Moraal 2013). Stronger solar activity is coupled to higher ϕ values. As the number of sunspots and the solar activity cycle ramps up, the amount of open solar flux (hereafter, OSF) also increases, and ϕ strengthens, while the HMF tilt also increases. These variations occur coherently, albeit with measurable physical time lags (Koldobskiy et al. 2022). Consequently, the cosmogenic isotope production rate decreases, establishing an inverse correlation with solar magnetic activity.

In addition to GCRs, Earth is sporadically bombarded by high-energy solar particles (solar energetic particles, SEP) that originate in reconnection processes at the Sun and are accelerated at flare sites, as well as at the shock-fronts of associated coronal mass ejections (CMEs). On average once every year a so-called ground level enhancement (GLE) event is registered on Earth: these are events of sufficient flux of energetic particles to be detectable with ground-based instruments such as neutron monitors (e.g. Simpson 2000). Recently, radionuclide records have revealed even greater events (Miyake et al. 2012). Largely because all other potential sources have been discounted, these Miyake events (ME) are generally thought to be extreme SEP events originating from the Sun, but their fluxes are several orders of magnitude greater than any SEP events that have been observed during the space era: hence these events are often also referred to as extreme solar particle events, (ESPEs, e.g., Usoskin et al. 2023). Six Miyake event candidates during the Holocene have been identified so far, several of which are confirmed across multiple cosmogenic records including ^10^Be, ^14^C, and ^36^Cl records, with the strongest events reported around 774/775 CE (Miyake et al. 2012) and 7176 BCE (Brehm et al. 2022). It is important to note that the inverse relationship between cosmogenic isotope production and solar magnetic activity breaks down during Miyake events, wherein the anomalous radionuclide spikes are produced not by modulation of the GCR flux, but by the direct bombardment of high-energy particles that are thought to be solar in origin.

Leveraging the aforementioned causal relationship between the HMF and GCR fluxes, long-term solar magnetic activity has been reconstructed using cosmogenic isotopes. Such studies range from establishing the background energetic particle variation over decadal timescales onto which Miyake events are added (Stuiver and Braziunas 1993; Miyahara et al. 2004; Fogtmann-Schulz et al. 2017) to millennial-scale solar magnetic activity reconstructions at annual resolution (Usoskin et al. 2021, 2025, 2026) and at coarser, decadal resolution (Stuiver and Braziunas 1989; Beer et al. 1990; Usoskin et al. 2003; Solanki et al. 2004; Vonmoos, Beer, and Muscheler 2006; Muscheler et al. 2007; Delaygue and Bard 2011; Steinhilber et al. 2012; Usoskin et al. 2016; Wu et al. 2018b).

While recent advances in multi-millennial SN reconstructions with improved annual resolution have significantly contributed to a better understanding of past space climate, they can produce unphysical results. The reconstructions are based on empirical formulation-based statistical regressions and a variety of errors can occur, even when the correlation coefficient associated with the regression is very high, as demonstrated by Lockwood et al. (2016). Causes can include non-linearities in the data, zero-level offsets, heteroskedasticity (parameter variance varying with amplitude), and non-normal distibution of fit residuals (tested for using quantile – quantile, “Q – Q”, plots). All of these can lead to erroneous regression and uncertainty parameters. In particular, when the regressions are applied outside the observed parameter range, such as during grand minima of solar activity, they can yield negative SNs. For these negative values the uncertainty will extend to, or over, the zero value if the uncertainty has been accurately estimated (Usoskin et al. 2021, 2025, 2026). As reconstructions of SN during grand minima are of particular interest for stellar dynamo and terrestrial climate research, a new methodology capable of reducing the uncertainties at these low activity is of great importance.

To address this, we develop a new stochastic forward search-based statistical inversion framework to infer the best (most plausible) temporal evolution of solar magnetic parameters from an arbitrarily long ϕ record. The temporal resolution of the reconstruction is limited by that of the ϕ dataset. Recent progress in the precise measurement of millennial-scale annual ϕ variation based on ^14^C in tree-rings (Brehm et al. 2021, 2025; Wang et al. 2026) enables us to derive an annually resolved reconstruction of SN over the past millennia using the new inversion technique. Instead of approximating the inverse physical mappings with empirical relations, the prescribed methodology combines data-informed priors on solar cycle properties with a sequence of forward models (Owens et al. 2024a,b) linking sunspot number to open solar flux and, subsequently, to heliospheric modulation. The inverse problem is then addressed using approximate Bayesian computation (ABC), wherein large ensembles of solar cycle realisations are generated, forward modelled, and evaluated against observed modulation potential without requiring an explicit likelihood function (Sisson, Fan, and Beaumont 2018; Beaumont 2019). The resulting posterior ensemble provides probabilistic estimates of sunspot number and of open solar flux that explicitly capture uncertainty and non-uniqueness. This approach produces physically admissible SN reconstructions, while artefacts such as negative SNs are self-consistently avoided, without requiring further post-processing of the reconstructions. All the model estimates of SN and OSF variations that we obtain with our methodology are uncertainty-quantified. We compare our reconstructed time series of sunspot number with direct observations during the telescopic era and show strong correspondence within uncertainties. The same is true for our OSF reconstruction which is compared with the most recent estimation by Owens et al. (2024a) based on four long geomagnetic data series. We also present a comparison of our reconstruction with the existing annual reconstruction over the past millennium (Usoskin et al. 2021).

The ϕ datasets used in the present work and their cross-calibration are described in Section 2. Section 3 presents an overview of the forward models, and Section 4 explains the method of inversion. Results are summarised in Section 5, followed by discussions and conclusions in Section 6.

## Heliospheric Modulation Potential Datasets

**Figure 1:**
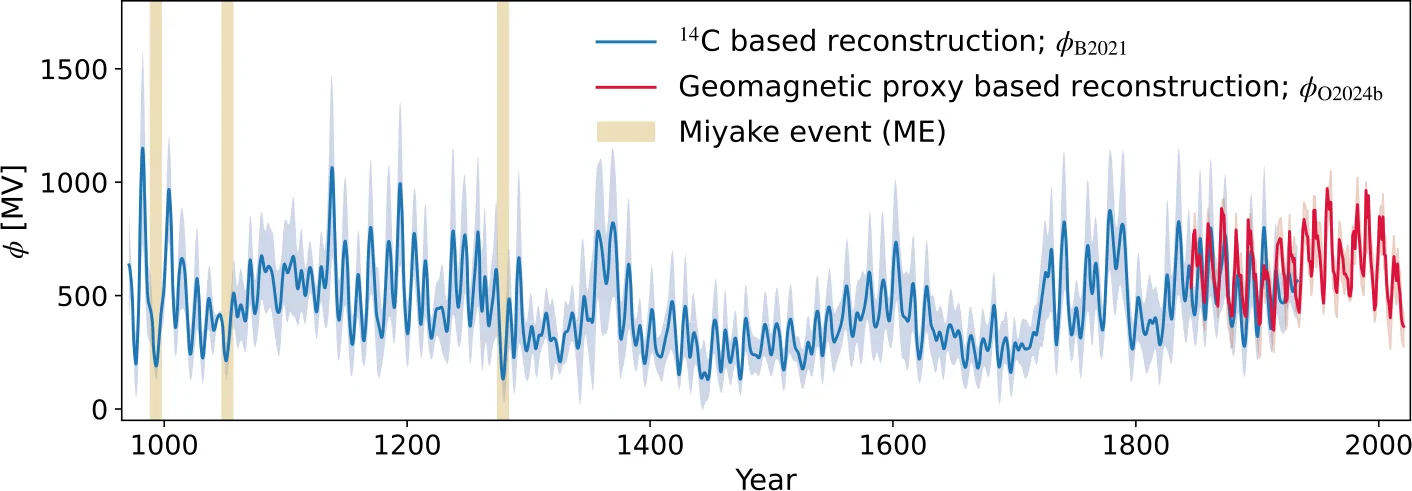
Estimates of the heliospheric modulation potential, ϕ, covering the past millennium: (i) $\phi _{B2021}$, based on cosmogenic ^14^ C based reconstruction (blue) by Brehm et al. (2021), and (ii) $\phi _{O2024b}$, computed from geomagnetic observations of open solar flux (red), by Owens et al. (2024b). Associated uncertainties are marked as shaded bands. These two datasets overlap for nearly eight solar cycles, during 1845 – 1932 CE. Three yellow-shaded intervals mark ± 5-year windows around the three proposed Miyake events (ME) in 993, 1052, and 1279 CE, respectively (Miyake, Masuda, and Nakamura 2013; Brehm et al. 2021).

Figure 1 illustrates two overlapping records of heliospheric modulation potential, ϕ – both at annual resolution – that we separately use as inputs in our inversion framework for reconstructing sunspot numbers. The shorter, more recent dataset spanning the period 1845 – 2023 CE is estimated from geomagnetic observations of OSF by (Owens et al. 2024b, hereafter, $\phi _{\mathrm{O2024b}}$). The longer dataset (Brehm et al. 2021, hereafter, $\phi _{\mathrm{B2021}}$) is reconstructed from cosmogenic ^14^C isotope records over the period 971 – 1932 CE. Notably, $\phi _{\mathrm{B2021}}$ does not incorporate the corrections for the Miyake events (Miyake, Masuda, and Nakamura 2013; Brehm et al. 2021) in 993, 1052, and 1279 CE. We use “MV” as the unit of ϕ throughout (see, Herbst et al. 2010; Beer, McCracken, and von Steiger 2012, for detailed discussions on the unit of the modulation function and the modulation potential). The combined record of ϕ demonstrates a wide range of solar activity, including both regular cycles, as we have experienced over the last century, and extreme phases over the past millennium.

**Figure 2:**
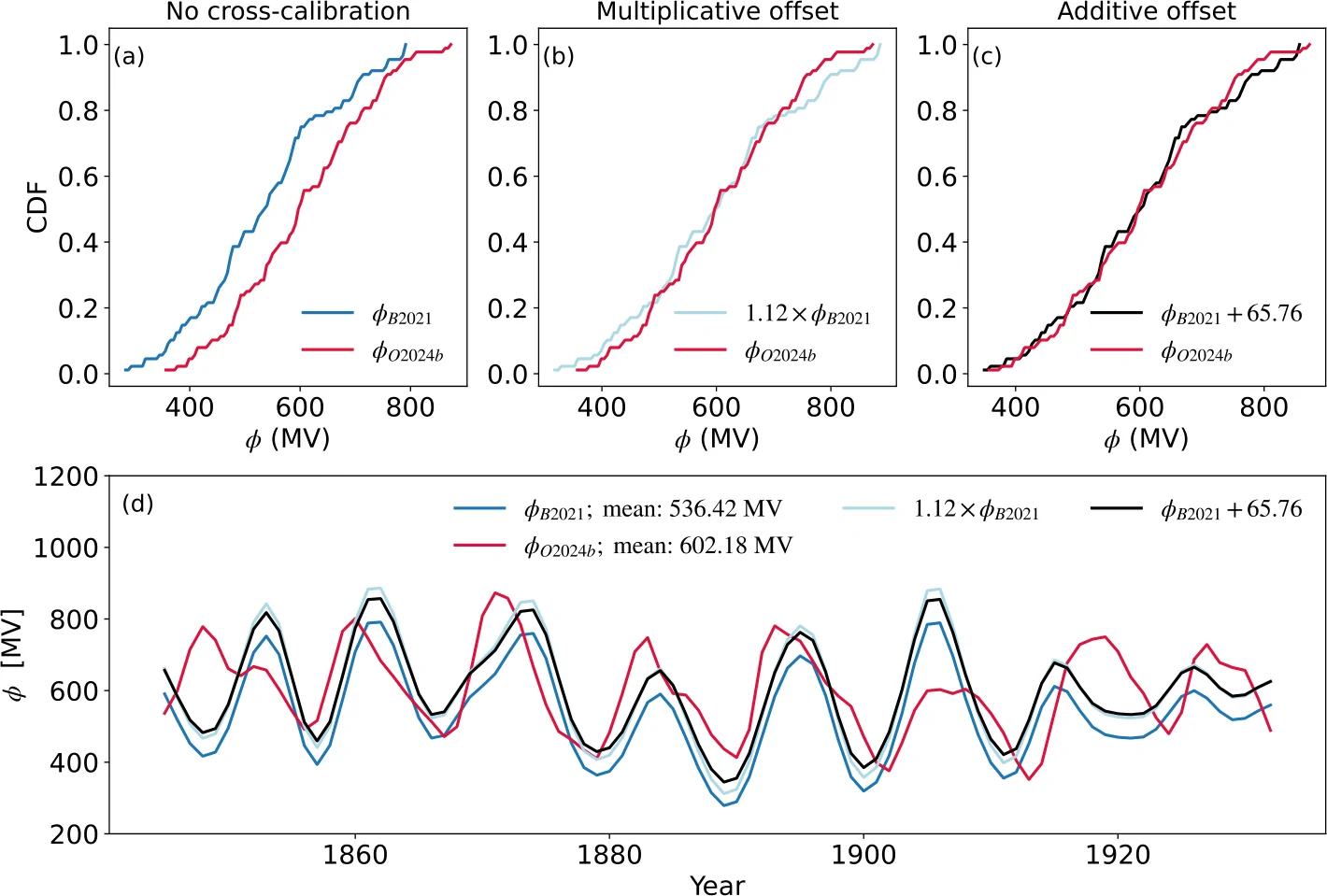
Cross-calibration of the two ϕ datasets across their overlapping interval 1845 – 1932 CE, to correct for their mutual offset as evidenced in their cumulative distribution functions (CDF, panel a). Considering $\phi _{O2024b}$ as the reference, we introduce multiplicative and additive offsets separately to $\phi _{B2021}$. Resulting CDFs are shown in the top panels b – c, and the time series are summarised in the bottom panel d. In our subsequent analysis with $\phi _{B2021}$, we apply an additive bias factor of 65.76 MV to the whole dataset, and refer to this corrected record as $\phi ^{*}_{B2021}$.

These two ϕ datasets exhibit a small systematic offset over their overlapping interval of 1845 – 1932 CE, as evidenced by the separation in their cumulative distribution functions (Figure 2a). This offset most plausibly arises from the fundamentally different measurement chains underlying each dataset. $\phi _{B2021}$ is inferred from atmospheric ^14^C concentrations recorded in tree rings, a process mediated by carbon cycle dynamics, geomagnetic field reconstructions, and cosmic ray production models, each of which carries its own systematic uncertainties. Rather than attempting to attribute the offset to any single physical source – which would require independent constraints not available here – we adopt an empirical cross-calibration over the overlapping interval. Treating $\phi _{O2024b}$ as the reference, on the grounds that it is derived more directly from heliospheric observations and yields excellent agreement with the instrumental sunspot record when used as input in our inversion model (see Section 5 for details), we compute the mean difference between the two datasets over their overlapping interval and apply this as a constant additive correction of 65.76 MV to the entirety of $\phi _{B2021}$. The corrected $\phi _{B2021}$ is hereafter referred to as $\phi ^{*}_{B2021}$. An additive rather than multiplicative correction is preferred because it achieves superior alignment of the full cumulative distribution functions across the overlapping period (see Figure 2b – 2d).

**Figure 3:**
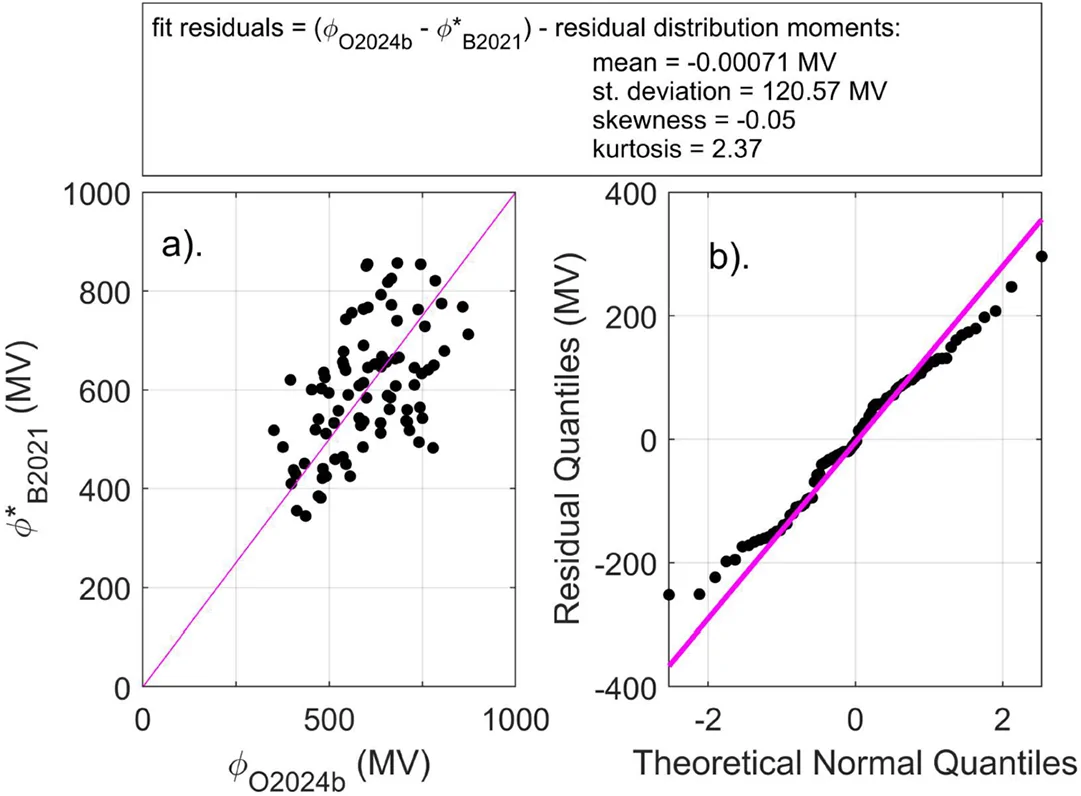
Confirmation of the validity of using the modulation parameter derived from ^14^C by Brehm et al. (2021), with offset correction, $\phi ^{*}_{B2021}$, to extend the series computed from geomagnetic observations of open solar flux by Owens et al. (2024b). $\phi _{O2024b}$. Panel a shows the scatter plot of $\phi ^{*}_{B2021}$ against $\phi _{O2024b}$ and reveals almost no heteroskedasticity. Panel b shows a quantile-quantile (Q – Q) plot of the fit residuals $\phi _{O2024b}-\phi ^{*}_{B2021}$ against the corresponding normal distribution.

Figure 3a shows a scatter plot of the modulation parameter derived from ^14^C by Brehm et al. (2021), with the offset correction derived in Figure 2, $\phi ^{*}_{B2021}$, against the simultaneous value computed from geomagnetic observations of open solar flux by Owens et al. (2024b), $\phi _{O2024b}$. The plot shows minimal “heteroskedasticity” (increase in variance with amplitude of either parameter), which is important because with heteroskedasticity, extrapolation becomes unreliable as the model incorrectly assumes constant variance, leading to systematically wrong confidence and prediction intervals, especially outside the observed data range; however, this scatter plot shows these data are highly “homoskedastic”. This can be quantified: dividing the dataset into three equal-size subsets by the $\phi _{O2024b}$ value, the standard deviations for the lower, middle, and upper tertiles are 51.82 MV, 34.86 MV, and 49.79 MV in $\phi _{O2024b}$ and 98.52 MV, 118.47 MV and 112.51 MV in $\phi ^{*}_{B2021}$. Hence there is no trend in the variance of either parameter. Note that using a multiplication correction to $\phi ^{*}_{B2021}$ would have acted to generate a trend and increase the heteroskedasticity. Panel b shows a quantile-quantile plot to compare the distribution of fit residuals $\phi _{O2024b}-\phi ^{*}_{B2021}$ to a normal distribution. The moments of the residual distribution are given in the top panel. The residual distribution mean is very close to zero showing the zero offset used is correct. The residual standard deviation means that the prediction accuracy is typically 120.57 MV. The skewness is −0.05. This very low value is extremely important and shows the residual distribution is almost perfectly symmetric and Gaussian-like in shape. Lastly the kurtosis (sharpness of the peak) is 2.37; this would be 3.00 if the distribution were normal. The smaller value than 3 reveals a slightly light-tailed distribution compared to a normal distribution, i.e. the residuals have fewer extreme outliers (either large or small) than a Gaussian model expects. This means any statistical test based on a normal distribution will be slightly conservative and also means that low values will not tend to be underestimates and that high values will not tend to be overestimates.

We acknowledge that assuming stationarity of the same additive offset across the full millennium is a simplification. Systematic biases in the carbon cycle model or geomagnetic field reconstruction may themselves vary with solar activity state or on centennial timescales, introducing uncertainties that are not captured by the correction applied here. The present correction should not, therefore, be regarded as a unique calibration.

## Forward Model Components

**Figure 4:**
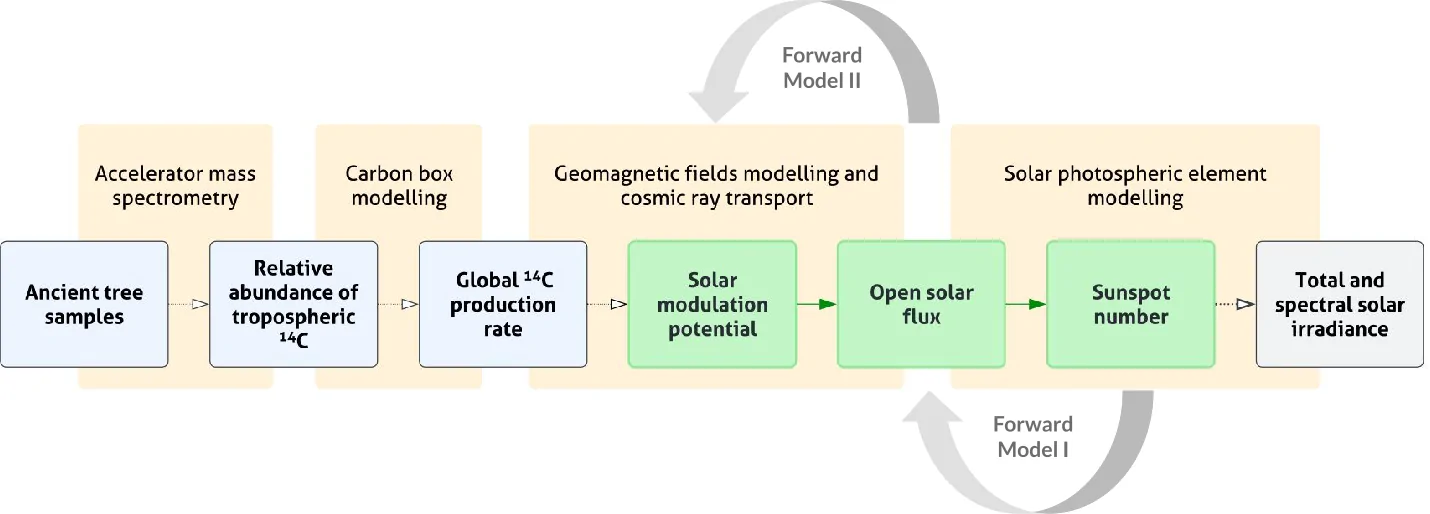
A schematic representation of the inversion sequence starting from dendrochronology to solar irradiance modelling via intermediate stages, as described in Section 1. Notably, paleoclimatic records of ^10^Be obtained from polar ice sheets complement ^14^C records at longer timescales. The present work involves only a part of the sequence marked in green boxes and with filled arrows, and it employs two deterministic forward models, I and II, as denoted by two curved arrows.

We employ a two-step forward modelling to derive ϕ from SN via an intermediate estimate of OSF, as part of the inversion sequence illustrated in Figure 4. This section outlines the forward models used within the overarching inversion framework.

### Defining a Template Sunspot Cycle

Our forward modelling begins by generating an ensemble of trial sunspot cycle realisations based on a set of statistical priors. Each trial realisation comprises one or more consecutive solar cycle segments spanning the time window of interest. These segments are synthesised from a representative solar cycle profile by scaling its amplitude and duration and by shifting the cycle start time.

**Figure 5:**
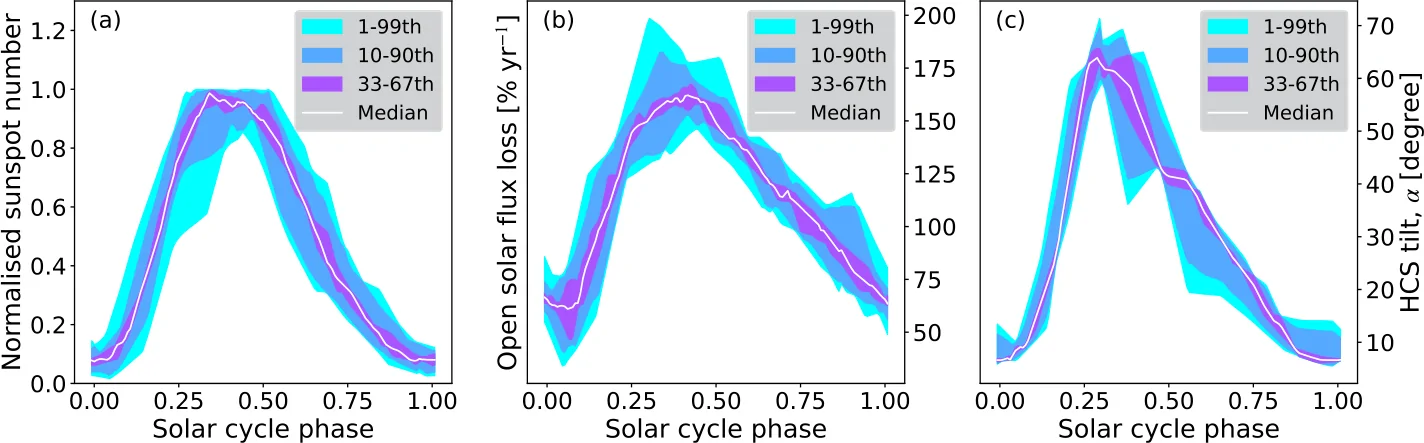
Phase-folded templates of sunspot cycle, SN(t); open solar flux loss rate, χ(t); and heliospheric current sheet tilt, α(t), in panels a – c, respectively. These temporal profiles are estimated from observations of long-term solar magnetic variability (see Owens et al. 2024a,b). In each panel, the cycle-averaged variation is shown in white, while coloured bands represent the 99%, 90%, and 67% of values. The cycle-averaged profiles are used as inputs to the forward models.

A wide range of empirical formulations has been proposed to parameterise the shape of solar cycles (Stewart and Panofsky 1938; Hathaway, Wilson, and Reichmann 1994; Sabarinath and Anilkumar 2008; Volobuev 2009; Du 2011; Li et al. 2017; Takalo and Mursula 2018). Here, we adopt the method of Owens et al. (2024a), which constructs a representative cycle from cycle-averaged sunspot numbers expressed as a function of solar cycle phase, derived from historical Solar Cycles 13 – 24. The resulting average profile is normalised to unit amplitude and unit phase length to produce the representative solar cycle template (see Figure 5, panel a). For any specified cycle duration, this phase-based template can be mapped directly to a temporal profile.

It is important to recognise that the template sunspot cycle does not preserve the Waldmeier effect (Hathaway 2015; Jaswal, Saha, and Nandy 2023), as any such correlation between sunspot cycle rise time and its amplitude is lost during the averaging and rescaling. However, for reconstructions on centennial to millennial timescales, this intra-cycle variability is not expected to significantly affect the inferred long-term behaviour but may shift the times of some peaks. There are ways in which the Waldmeier effect could be incorporated but for the sake of simplicity we here do not include it. Section 4 details the statistical priors used to generate the full ensemble of solar cycle realisations from this representative template.

### Forward Modelling Sunspot Numbers to Open Solar Flux

The first model (i.e., Forward Model I in Figure 4) in the forward model sequence computes the total unsigned OSF from the time-varying SN. A consistent and measurable definition of OSF is that it is the magnetic flux that threads a hypothetical heliocentric surface – known as the source surface – located near the outer boundary of the solar corona (Lockwood and Owens 2014). At this spatial scale, the individual contributions from discrete photospheric magnetic elements are no longer significant (Hore and Bhowmik 2026), allowing OSF to be treated as a global quantity. The temporal evolution of OSF can therefore be described using a global equation of balance, an approach introduced by Solanki, Schüssler, and Fligge (2000) and subsequently refined in later studies (Lockwood 2003; Owens and Crooker 2006, 2007; Vieira and Solanki 2010; Schwadron, Connick, and Smith 2010; Owens, Usoskin, and Lockwood 2012; Goelzer, Smith, and Schwadron 2013; Krivova et al. 2021; Owens et al. 2024a).

In this work, we model the OSF evolution at time t as follows,

1 $$ \dfrac{d \mathrm{OSF}(t)}{dt} = S(\mathrm{SN},t) - \chi (t) \mathrm{OSF}(t), $$

with an SN dependent source term, $S(\mathrm{SN},t)$, and a data-driven, nonlinear flux loss term, $\chi (t) \mathrm{OSF}(t)$. The emergence of photospheric magnetic structures, including sunspots, acts as a source of OSF through the resulting solar wind outflow and CMEs (and, potentially, other field loops) transporting new closed magnetic flux into the heliosphere. OSF is assumed to be lost through reconnection below the source surface. We assume a cycle-invariant fractional OSF loss rate, χ(t), as a function of solar cycle phase, as found by Owens and Lockwood (2012) (see Figure 5, panel b). The profile of χ(t) is taken as the cycle-averaged mean OSF loss rate derived from historical Solar Cycles 13 – 24 (see Owens et al. 2024a, for further details).

To calculate the source function from SN, we use the following empirically optimised formulation prescribed by Owens et al. (2016, 2024a),

2 $$ S(\mathrm{SN}, t) = 0.84\,[\mathrm{SN}(t) + 2.67]^{0.54} \, - \, 0.0055 \quad \quad [\times 10^{14}\,\, \mathrm{Wb} \, \mathrm{yr}^{-1}], $$

For a given sunspot cycle realisation, $\mathrm{SN}(t)$, the source function is calculated using Equation 2, which is then fed into Equation 1. We solve Equation 1 numerically using a forward-difference scheme with an annual timestep and a prescribed initial value for the OSF, $\mathrm{OSF}(0)$. This is estimated from SN at that timestep by assuming a first-order approximated linear SN-OSF relationship,

3 $$ \mathrm{OSF}(0) = 0.034\, \mathrm{SN}(0) + 4.208 \quad \quad [\times 10^{14} \,\, \mathrm{Wb}], $$

which is derived from a linear regression between the geomagnetic OSF record presented in Owens et al. (2024a) and the SILSO sunspot number record (Clette and Lefèvre 2015) over the corresponding period of 1845 – 2020 CE. The influence of this initial value dissipates within a few timesteps and does not greatly affect the resulting OSF time series. Therefore, an accurate estimation of initial OSF is not critical in this case.

### Forward Modelling Open Solar Flux to Heliospheric Modulation Potential

The estimated open solar flux is passed to another forward model (i.e., Forward Model II in Figure 4) that computes the heliospheric modulation potential, ϕ. The heliospheric modulation potential is an estimate of the effective energy loss per nucleon experienced by GCRs as they propagate through the heliosphere. This energy loss arises from transport processes including diffusion, advection by the solar wind, particle drifts, and adiabatic deceleration (Parker 1965). Thus, ϕ provides a useful proxy for the large-scale strength and structure of the heliospheric magnetic field (Asvestari and Usoskin 2016; Owens et al. 2024b). Whereas the strength of HMF can be quantified in terms of OSF, its structure is governed by the tilt and polarity of the heliospheric current sheet (HCS).

Following Owens et al. (2024b), we calculate ϕ as,

4 $$ \phi (\mathrm{OSF}, \alpha , p^{*}) = \phi _{0} \ {\mathrm{OSF}}^{n} \ (1 + A \sin \alpha ) \ (1 + B p^{*}), $$

where $\phi _{0}$, A, B, and n are four optimised free parameters with the values 647 MV, 0.404, − 0.0742, and 0.594, respectively (see Owens et al. 2024b, for details). The observed HCS tilt is denoted by α, and p∗ denotes its effective polarity. Notably, this formulation allows ϕ to modulate significantly even with negligible OSF – this is particularly relevant for explaining the persistence of solar magnetic variability during grand solar minima phases (Beer, Tobias, and Weiss 1998; Miyahara et al. 2004; Charbonneau, Blais-Laurier, and St-Jean 2004; Owens, Usoskin, and Lockwood 2012; Saha, Chandra, and Nandy 2022; Dash, Nandy, and Usoskin 2023).

The effective polarity $p^{*}$ is defined in terms of effective HCS tilt angle $\alpha ^{*}$ and the HMF polarity p,

5 $$ p^{*}=p(1-\sin \alpha ^{*}), $$

where p assumes values ±1 depending on the solar cycle phase. The effective tilt $\alpha ^{*}$ rescales the observed HCS tilt α such that it spans $0^{\circ}$ to $90^{\circ}$ in each solar cycle,

6 $$ \alpha ^{*} = \frac{\pi}{2}\left ( \frac{\alpha -\alpha _{\mathrm{MIN}}}{\alpha _{\mathrm{MAX}}-\alpha _{\mathrm{MIN}}} \right ), $$

Based on cycle-averaged HCS tilt measurements, Owens et al. (2024b) reported $\alpha _{\mathrm{MIN}}= 8^{\circ}$ and $\alpha _{\mathrm{MAX}}=61^{\circ}$. Similar to the template phase profiles for the sunspot cycle and the OSF loss function, a representative profile for the HCS tilt variation is derived from averaging over the observations of Solar Cycles 20 – 24 (see Figure 5, panel c).

## The Bayesian Inversion Through ABC Rejection Sampling

The solution of an inverse problem – such as the one considered in this work – involves reconstruction of the underlying causes from observed effects. Here, we infer sunspot numbers (the cause) from historical observations of the heliospheric modulation potential (the effect). At any given time, the estimation of OSF and hence ϕ depends not only on the contemporaneous solar activity but also on its prior history, which introduces hysteresis into the SN-OSF-ϕ relationship (Owens et al. 2024a). This OSF hysteresis renders the inverse mapping non-local in time and non-unique, i.e., multiple, physically distinct SN histories can produce statistically indistinguishable ϕ time series. Consequently, even small measurement uncertainties in the effect can amplify during reconstruction, leading to unphysical solutions. Therefore, the reconstruction of SN from ϕ is an ill-posed problem and may not be reliably solved by direct, deterministic inversion techniques.

Bayesian inversion offers a principled approach for addressing non-uniqueness by combining prior information with observational constraints. However, a conventional Bayesian formulation would require specification of a likelihood function linking modelled and observed ϕ, as well as informative priors and their temporal covariance (Gelman 2013). For pre-telescopic periods and extended intervals of low solar activity, such priors are poorly constrained by independent observations (Muñoz-Jaramillo and Vaquero 2019) and may therefore dominate the posterior distribution. Furthermore, the strong hysteresis inherent in OSF evolution may lead to highly multimodal and irregular likelihood surfaces, significantly complicating convergence and interpretation of standard Bayesian sampling schemes.

To overcome these limitations, we adopt an ABC-based approach implemented via rejection sampling (ref. Sisson, Fan, and Beaumont 2018, for an introduction). ABC avoids the need to calculate an explicit likelihood function, replacing it with a simulation-based rejection criterion (cf. Beaumont 2019). Model parameters are drawn from specified prior distributions, are propagated through the complete forward model chain, and are kept if the simulated output agrees with the observed data within an allowable tolerance. Therewith, the collection of accepted simulations in the space of the distance metric approximates the posterior distribution, offering a clear depiction of uncertainty and non-uniqueness.

### Windowing

We adopt a windowed Bayesian inference approach, resolved at the level of individual sunspot cycles. This approach estimates local posteriors over short intervals rather than a single global posterior across the full dataset. Each window defines the local data context for cycle-level inference, while priors on cycle parameters are weakly informed by the statistical properties of the observed sunspot record. Thus, it avoids any assumption of global stationarity by treating each cycle as a distinct realisation, with parameters that evolve gradually over millennial timescales.

The window length is a free parameter of the model that is fixed globally at the start of an inversion run and remains constant throughout the inference from the full input dataset. A shorter window contains fewer cycles and results in a lower-dimensional prior and posterior space, while a longer window increases the dimensionality, and hence increases the computational cost. To put this into perspective, for N cycles within a window, the prior space is (2N+1)-dimensional, with one amplitude and one duration parameter per cycle plus a single start-offset parameter. A practical lower bound on the window length is set by the finite OSF memory. In the present work, we optimally use window lengths of 10 and 15 years as stated in the captions of Figures 8 and 9, respectively.

To account for the finite memory inherent in OSF evolution, the inversion is performed within overlapping temporal windows. In the absence of any source term in Equation 1, the inverse of the OSF loss rate, i.e., the e-folding timescale determines the OSF memory. The loss rate, χ(t), varies within 50 – 200% per year in each cycle (see Figure 5b), thus the OSF memory typically retains for 0.5 – 2 years, which is well below the chosen window length.

Adjacent windows are offset by a 1-year stride. The inference within each window is entirely local and independent, i.e., the posterior from one window does not influence the prior of the next. For a window of length L years advanced with a stride of 1 year, successive windows overlap by (1−1/L)×100% of their length. This means each year in the reconstruction is covered by multiple overlapping windows, each with its own posterior estimates. These multiple estimates for a year are combined into a single annual value by computing a weighted average, where the weight assigned to each window is inversely proportional to its mean absolute error against the target ϕ data over that specific window. The stride length governs the degree of overlap between successive windows, e.g., a shorter stride produces more overlapping estimates per year and hence a smoother composite reconstruction.

### Distributions of Priors

Three morphological parameters of individual sunspot cycles – amplitude, duration, and start offset – can sufficiently define the SN timeseries realisations within each window. Large Monte Carlo (MC) ensembles of these parameters are sampled from their corresponding prior distributions.

The priors are broad and weakly informative, spanning the full range of solar cycle variability observed during the telescopic era while avoiding restrictive assumptions about cycle-to-cycle correlations, as the validity of these relations in the past is unknown.

Cycle amplitudes are sampled from a log-normal distribution with unit standard deviation, chosen to accommodate the strictly positive and right-skewed nature of the SN distribution while remaining sufficiently broad to encompass episodes of both weak and strong solar activity. For a given window, the mean cycle amplitude, $\langle SN \rangle _{\mathrm{window}}$, is guided by the mean heliospheric modulation potential, $\langle \phi \rangle _{\mathrm{window}}$, through the following regression,

7 $$ \langle SN \rangle _{\mathrm{window}} = 0.5 \langle \phi \rangle _{ \mathrm{window}} - 246.8 $$

The above relation is empirically estimated from instrumental-era records of SN (Clette and Lefèvre 2015) and ϕ (Owens et al. 2024b). To ensure physically admissible cycle amplitudes during extended low-activity intervals, the mean amplitude prior is restricted to non-negative values, for which the log-normal distribution would be valid. It is important to note that this mean value is used solely to improve sampling efficiency by localising the prior over the region of parameter space most compatible with the observed activity level. It does not impose a deterministic constraint on the reconstructed SN.

Priors for cycle lengths are drawn randomly from a Gaussian distribution with a mean and standard deviation of 10.5 years and 2 years, respectively. Motivated by historical SN observations, the distribution is truncated at 7 years and 15 years.

The start offset of the first cycle in each window is sampled from a uniform distribution between − 11 and 0 years. Here, a value of −X years indicates that the first sunspot cycle within the window is already X years into its progression from the preceding minimum at the time the window starts.

Together, these distributions define a set of priors that broadly assimilate the statistical characteristics of the observed solar cycle record. Each MC draw generates a realisation of solar cycle evolution within a window using the representative cycle template described in Section 3.1. The left panel of Figure 7 illustrates the prior parameter distributions for a representative window, while the right panel shows the corresponding posterior distributions inferred through forward modelling and ABC rejection sampling.

### The Error Metric

Each trial sunspot cycle realisation covering a specific window is propagated forward through the coupled SN–OSF–ϕ model to generate an ensemble of MC-simulated heliospheric modulation potential, $\phi _{\mathrm{MC}}(t)$. The agreement between $\phi _{\mathrm{MC}}(t)$ and the observed modulation potential, $\phi _{\mathrm{target}}(t)$, is quantified using a weighted Euclidean distance (Prangle 2017) that measures the pointwise error

8 $$ \epsilon = \left [\sum _{i} \left ( \frac{\phi _{\mathrm{MC}}(t_{i}) - \phi _{\mathrm{target}}(t_{i})}{\sigma _{i}^{2}} \right )^{2}\right ]^{1/2}, $$

where $\sigma _{i}$ represents an estimate of the uncertainty in the target data at each timestamp, $t_{i}$, within the window. Rather than compressing the observed data into lower-dimensional summary statistics, we compare simulated and observed trajectories directly in this manner. This is equivalent to treating the full observed window as the sufficient statistic for the cycle parameters, an assumption that is reasonable when the forward models produce smooth synthetic cycles whose shape is directly governed by the parameters of interest.

In ABC rejection sampling, the acceptance of an MC realisation is conventionally determined by comparison of its error metric with a prescribed tolerance threshold, $\epsilon _{\mathrm{tol}}$. Realisations satisfying $\epsilon \leq \epsilon _{\mathrm{tol}}$ are retained as samples from the approximate posterior, while those exceeding the tolerance are rejected. However, a fixed tolerance implicitly assumes that the absolute discrepancy between simulated and observed summary statistics is comparable across all estimation windows. In a non-stationary setting such as millennial-scale solar activity, this assumption fails, i.e., windows during grand maxima exhibit higher absolute activity levels and larger natural variability, producing systematically larger discrepancies than windows during quiescent periods. A fixed $\epsilon _{\mathrm{tol}}$ therefore becomes either overly permissive during active epochs or prohibitively strict during quiet epochs, causing the effective sample size and approximation quality to vary arbitrarily across the time series.

**Figure 6:**
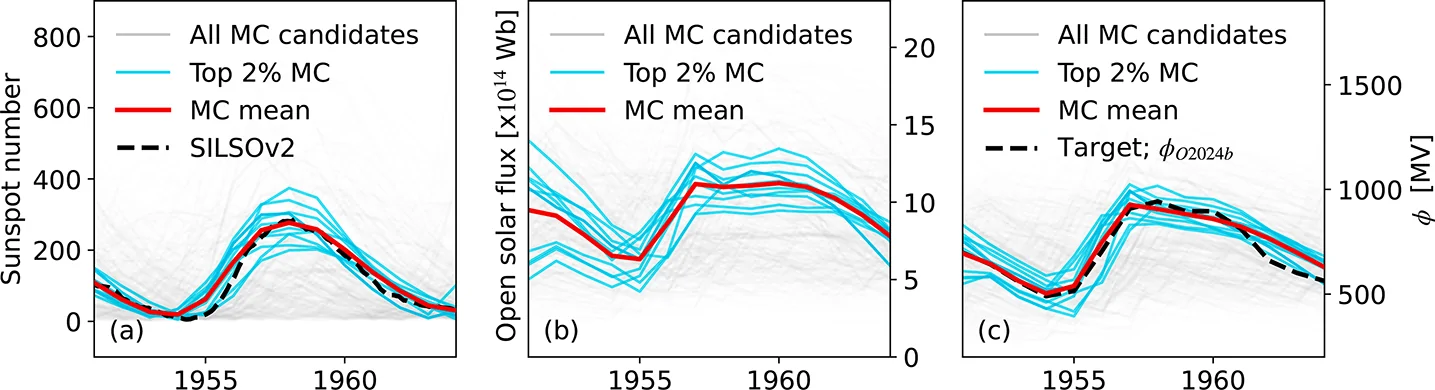
Example of the stochastic forward modelling sequence over an arbitrarily chosen time window during 1950 – 1965. The sequence starts with an ensemble of 500 MC-simulated sunspot cycle candidates (left panel), their corresponding estimates of OSF (middle panel), and ϕ (right panel). The top 2% of these MC realisations, ranked according to their agreement with the target modulation potential $\phi _{\mathrm{O2024b}}$ (black-dashed curve in panel c), define the ensemble of accepted solutions (in cyan). The arithmetic mean of these accepted realisations is adopted as the mean estimate (in red) over the selected window in each panel. Panel a overlays the best estimate of sunspot number (in red) on the directly observed sunspot number data from SILSOv2 (in black dashed curve, Clette et al. 2023) for the corresponding period.

To bypass this, we adopt an adaptive tolerance. We rank all MC simulated realisations in a window in ascending order of their associated error metric; i.e., the realisation with the least error receives the topmost rank, and so on (see Figure 6). From a large number of such MC realisations in each window – 10,000 in our case – we select the top 2% candidates and accept them as probable solutions. The choice of 2% acceptance is found to be sufficiently strict in the present context, with 200 accepted candidates forming the posterior space. This approach ensures a consistent approximation quality and, therefore, comparable posterior reliability across all windows.

### Estimation of the Posterior Credible Band

We consider the arithmetic mean of the accepted candidates, i.e., the best 2% of MC realisations, as the inferred SN, OSF, and ϕ in each window. To estimate the associated uncertainties, we report the 68% highest posterior density (HPD) interval (Box and Tiao 1965). The HPD is defined as the shortest interval containing a specified probability mass, with the property that every point inside the interval has a higher posterior density than every point outside it. For the posteriors arising from ABC inference on solar cycle parameters, symmetry is not guaranteed – particularly near grand minimum epochs, where the amplitude posterior is expected to be skewed toward low-activity values. In such cases, the equal-tailed interval can include parameter values of low posterior probability while excluding values of higher probability, producing a misleading representation of posterior uncertainty. The HPD interval avoids this by construction, always identifying the most credible region of parameter space regardless of posterior shape.

**Figure 7:**
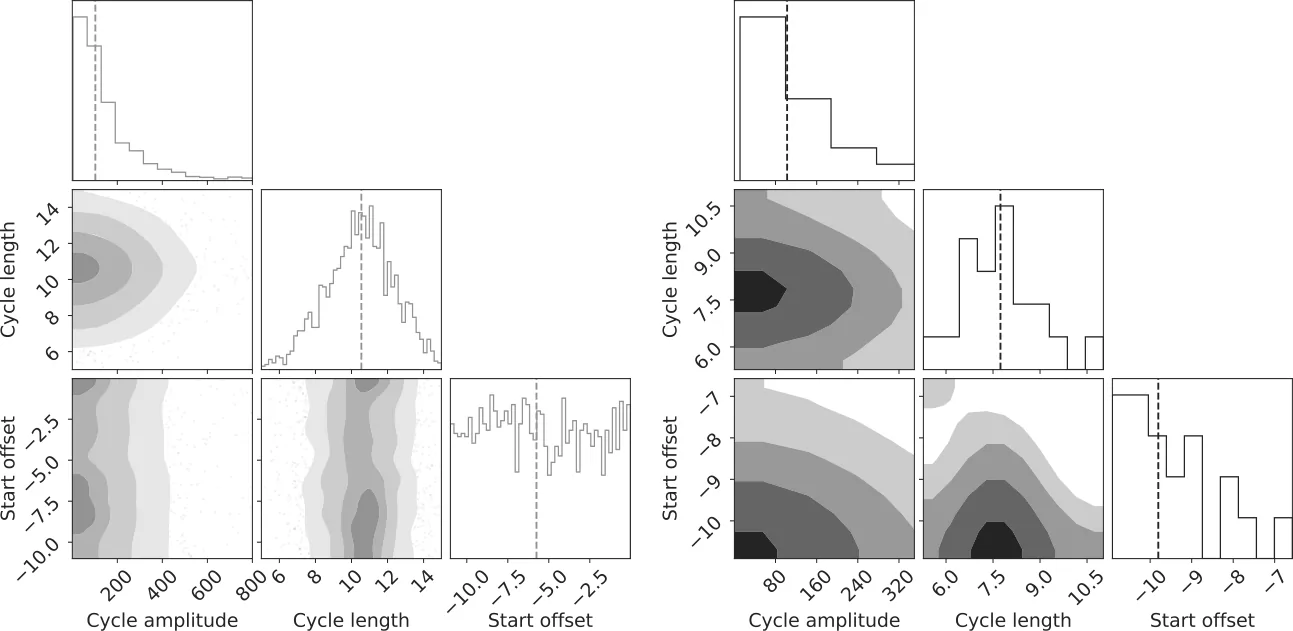
Corner plots showing the prior (left) and posterior (right) distributions of three model parameters, i.e., cycle amplitude, length, and start-time offset for an arbitrarily chosen sunspot cycle. Diagonal and off-diagonal panels show the marginal distributions and pairwise cross-correlations, respectively.

The present inversion technique is equivalent to ABC rejection sampling with a uniform kernel and an adaptively chosen tolerance. It provides a computationally efficient means of sampling the space of physically admissible solar histories without requiring an explicit likelihood function. Figure 7 illustrates the ABC rejection process, highlighting the contraction from the broad prior ensemble to a constrained set of posterior realisations consistent with the observed ϕ within uncertainty. Owing to the prescribed sunspot cycle shape and the imposed priors on cycle amplitudes, the reconstructed SN remains strictly non-negative and is therefore physically realistic.

The choices of window length, stride, and ensemble size represent a balance between computational tractability and adequate sampling of the posterior solution space. The resulting ensemble of accepted realisations provides a joint reconstruction of SN, OSF, and ϕ, with uncertainties quantified directly from the ensemble spread. The application of the current inversion method on different ϕ datasets and the benchmarking of their outcomes are presented in Section 5.

## Results

We apply the inversion method described above to two heliospheric modulation potential datasets, $\phi _{O2024b}$ (Owens et al. 2024b) and $\phi ^{*}_{B2021}$ (Brehm et al. 2021), respectively.

**Figure 8:**
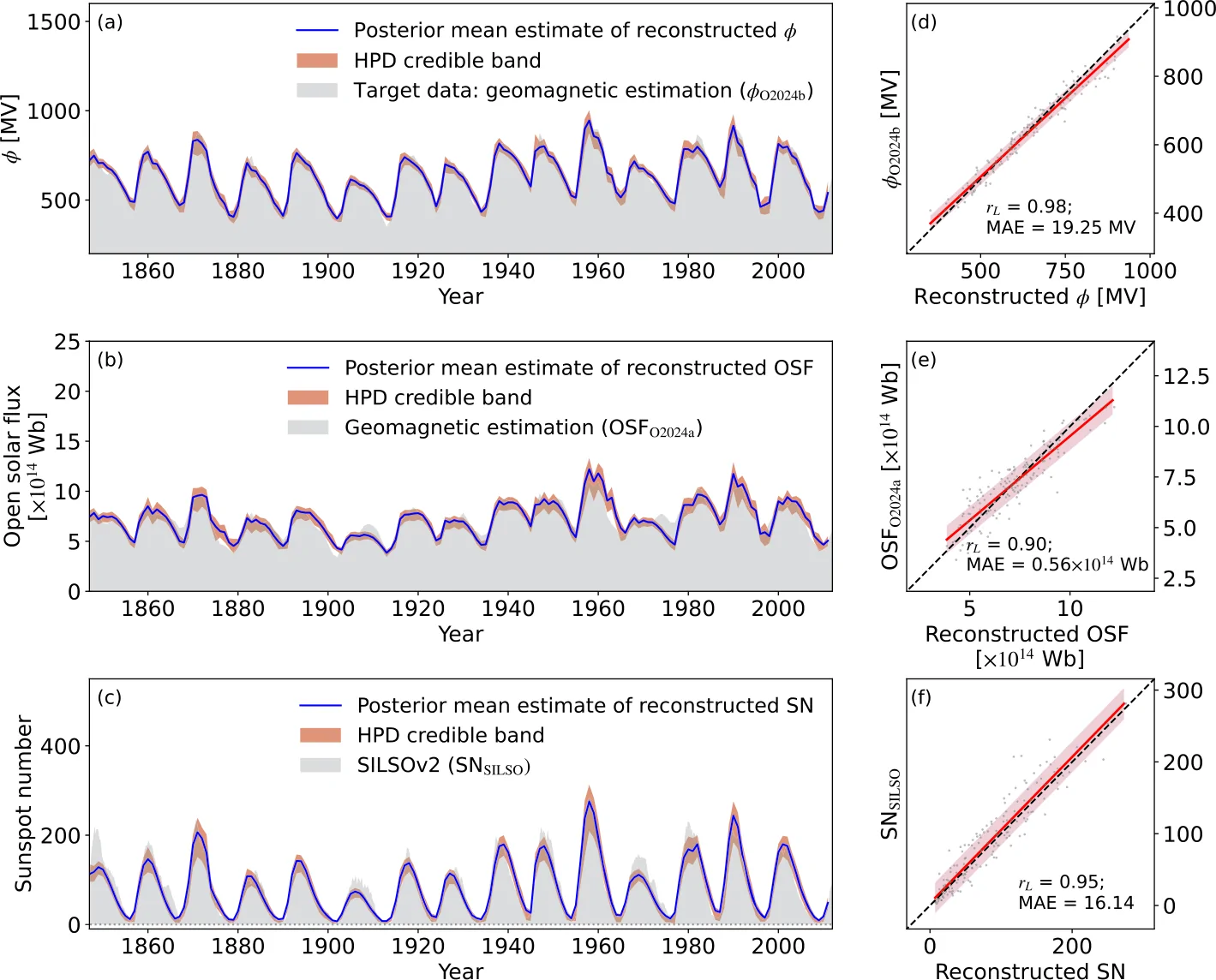
Evolution of annually resolved heliospheric modulation potential, ϕ (panel a), open solar flux (panel b), and sunspot number (panel c), reconstructed from geomagnetic estimate of ϕ (Owens et al. 2024b). The blue curves and pink shaded regions denote, respectively, these three physical quantities and their associated uncertainties inferred from the inverse modelling. Grey-shaded regions represent direct observations for comparison, namely, the geomagnetic estimate of ϕ (Owens et al. 2024b), open solar flux (Owens et al. 2024a), and the SILSOv2 international sunspot number record (Clette et al. 2023), in panels a – c, respectively. Panels d – f show strong linear correlations between reconstructed and observed values, along with the corresponding mean absolute errors (MAEs). Black dashed lines in panels d – f represent y=x. The window length for this set of simulations is set at 10 years.

Figure 8 shows the resulting reconstructions of open solar flux and sunspot number when $\phi _{O2024b}$ is used as the target in the MC simulations. The recovered ϕ yields a mean absolute error of 19.25 MV relative to $\phi _{O2024b}$, i.e., 3.06% of its mean value. Linear correlations between the reconstructed series and the corresponding observations are shown in panels d – f of Figure 8. All three reconstructions corroborate with direct space- and ground-based observations of open solar flux (Owens et al. 2024a) and sunspot number (Clette and Lefèvre 2015). The reconstructed open solar flux slightly but systematically underestimates weak fluxes and overestimates strong fluxes; however, the deviation remains within the associated uncertainties (see Figure 8, panel e). The overall agreement between the observed records and their reconstructed counterparts validates the performance of the inversion model. It justifies its applicability to older, longer records of heliospheric modulation potential.

Since the SN reconstruction based on $\phi _{O2024b}$ yields an excellent agreement with the SILSO sunspot record within the uncertainty, this provides an additional justification for considering $\phi _{O2024b}$ as a standard estimate and re-calibrate $\phi _{B2021}$ accordingly. Following the cross-calibration described in Section 2, we apply $\phi ^{*}_{B2021}$ as the target for the millennial-scale reconstruction.

**Figure 9:**
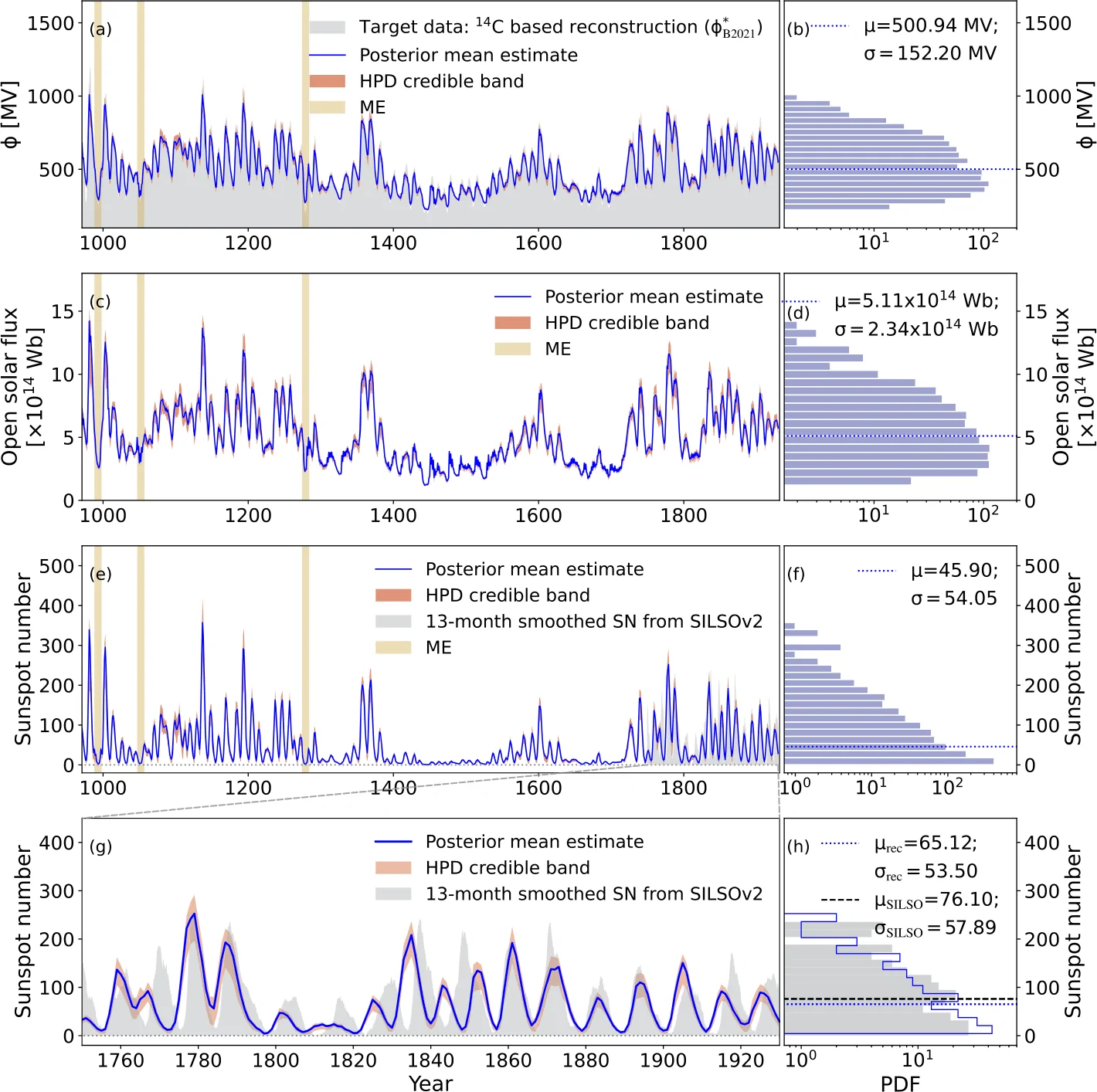
Millennial scale, annually resolved reconstructions of ϕ (panel a), open solar flux (panel c), and sunspot number (panel e and g) during 971 – 1932 inferred from ^14^C-based estimate, $\phi ^{*}_{B2021}$. The blue curve in each panel denotes the mean reconstruction, and the pink shades signify the uncertainty bands. Three yellow-shaded intervals in panels a, c, and e mark a ± 5-year window around the three proposed Miyake Events in 993, 1052, and 1279 CE, respectively (Miyake, Masuda, and Nakamura 2013; Brehm et al. 2021). Panels on the right show the corresponding probability distribution functions (PDF), along with the mean (μ) and standard deviation (σ). Panel g covers the period of directly-observed sunspot number data from SILSOv2 (Clette et al. 2023), with its corresponding PDF (grey shading) shown in panel h. Note that all the PDFs are in semi-logarithmic scale. The comparison in panels g and h indicates a small, persistent underestimation of sunspot numbers by the inference model throughout the overlapping window (see the text for discussions). The window length is increased to 15 years in the present case to better accommodate potentially longer cycles, if any, during pre-telescopic epochs.

Figure 9 summarises the reconstructions of heliospheric modulation potential, open solar flux, and sunspot number derived from $\phi ^{*}_{B2021}$. Panels a, c, and e show the reconstructed ϕ, OSF, and SN time series, respectively, along with their associated uncertainties. The corresponding probability distributions, including the mean and standard deviation, are illustrated in panels b, d, and f. The reconstructed SN remains strictly non-negative throughout the past millennium, as evident in both panels e and f in Figure 9. Panel g zooms into the recent interval overlapping with the SILSO record. In contrast to the reconstruction based on $\phi _{O2024b}$, the agreement between the reconstructed SN and the SILSO observations degrades in this case. This discrepancy is also apparent in the probability distributions shown in panel h, where the newly reconstructed mean SN ($\mu _{ \mathrm{rec}}=65.12$, blue dotted line) is lower than the SILSOv2 mean ($\mu _{\mathrm{SILSO}}=76.10$). For comparison, the U2021 (Usoskin et al. 2021) reconstruction yields a mean SN of 59.66, with a standard deviation, 62.73, computed over 1749 – 1899 CE, i.e., the period of overlap with the SILSOv2 record.

Despite the additive offset on $\phi _{B2021}$, there is a significant contrast in the performance of the same inversion methodology when $\phi ^{*}_{B2021}$ is used as the target instead of $\phi _{O2024b}$. In particular, the constant additive correction applied to $\phi _{B2021}$ may not fully account for activity-state-dependent biases in the carbon cycle or geomagnetic field reconstruction, such that the residual offset between the two datasets – and consequently the reconstructed SN – could vary systematically across different phases of solar activity, as acknowledged in Section 2.

This work sets the most meaningful (and lowest) value to date of the minimum value in annual mean of the (unsigned) OSF. From Figure 9 we see that the unsigned OSF fell to a value of $1.21^{+0.09}_{-0.10} {\times}10^{14}$ Wb in 1443 CE, during the Spörer minimum. The lowest value in the new reconstruction during the Maunder minimum is $1.73^{+0.19}_{-0.18}{\times}10^{14}$ Wb (in 1668 CE) and an average for the Maunder Minimum was about $2.63^{+0.16}_{-0.23}{\times}10^{14}$ Wb. The lowest geomagnetic OSF value in the data by Lockwood and Owens (2024) (which covers 1844 – 2023 CE) is $2.58{\pm}{1.17}{\times}10^{14}$ Wb for the Carrington Rotation (CR) 631 in 1900. Note that Lockwood and Owens 2024 derived the signed OSF which is half the unsigned values quoted in this paper. The present paper shows that extending the time series back to 971 CE generates lower values and that the lowest known annual mean value of the unsigned OSF is $1.21^{+0.09}_{-0.10}{\times}10^{14}$ Wb which is considerably lower than the lowest seen even during the Maunder minimum and nearly a quarter of the original postulate for an OSF floor by Svalgard and Cliver (2007). Given there is no theoretical expectation for this floor value, it remains possible that even lower values could have existed before the last millennium.

The average Maunder minimum value of the unsigned OSF of $2.63^{+0.16}_{-0.23}{\times}10^{14}$ Wb is significantly higher than the value of $0.96{\pm}0.58{\times}10^{14}$ Wb that was derived by simple linear regression using ^10^Be cosmogenic isotope data by Lockwood (2013) and slightly higher than the value of $2.03{\times}10^{14}$ Wb from the reconstruction by Usoskin et al. (2021). However it is slightly lower than the model prediction by Lockwood and Owens (2014) which yielded $(2.79{\pm}0.92{\times}10^{14}$ Wb. Nonetheless, there is considerable overlap between the uncertainty bands for these model predictions and the OSF reconstruction presented here which is encouraging as the modelling used the same OSF production and loss rates as the forward modelling of OSF from sunspot numbers deployed here. Hayakawa et al. (2021) noted a non-linear relationship between the peak sunspot group number, $R_{G}$ and the peak OSF which, when extrapolated to $R_{G} = 0$, yielded $3.13{\times}10^{14}$ Wb which is slightly greater than the uncertainty band derived here.

**Figure 10:**
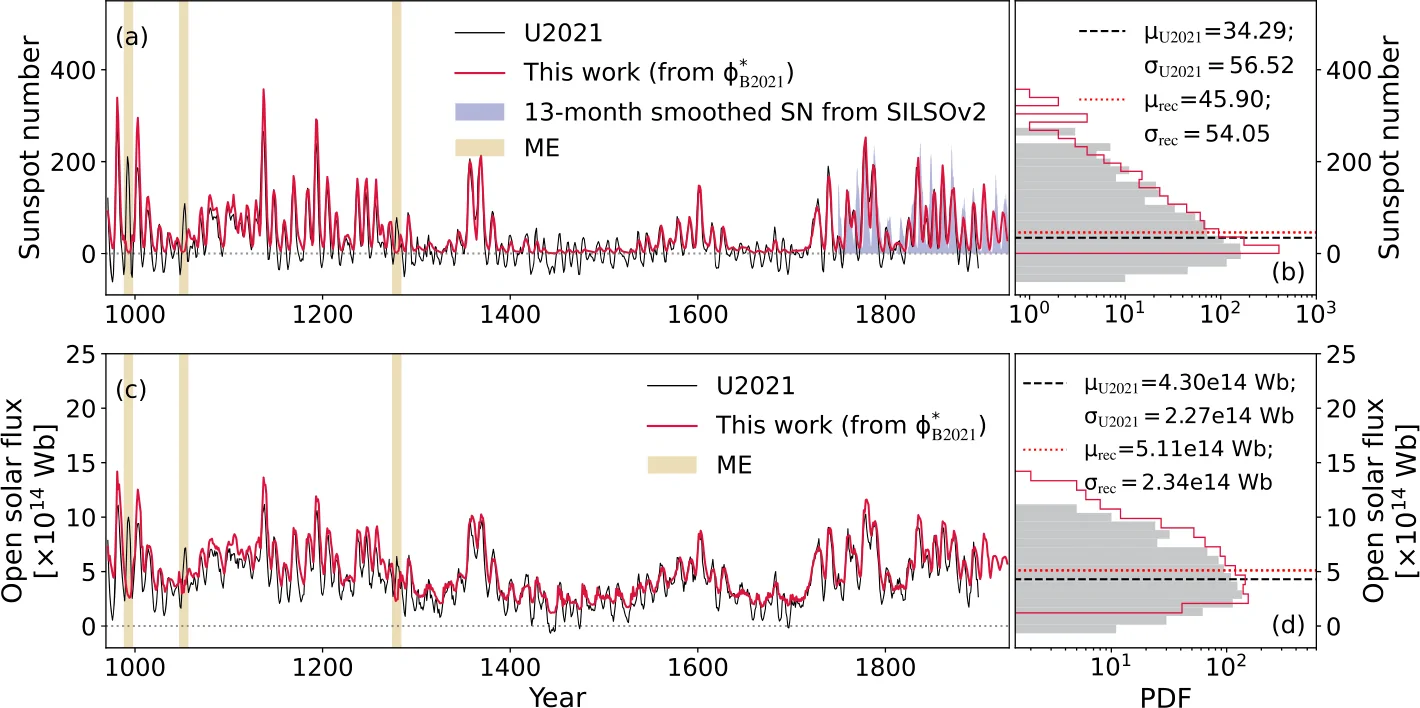
A comparison of the present reconstruction (red) of sunspot number (panels a, b) and open solar flux (panels c, d) with a previous reconstruction (black) by Usoskin et al. (2021). Since the parent ϕ dataset $\phi _{B2021}$ is not corrected for the proposed Miyake events in 993, 1052, and 1279 CE (see text), the current reconstruction might miss three sunspot cycles around those events, as highlighted in the yellow shaded intervals. Hence, the present reconstruction within these intervals might not be reliable.

Figure 10 compares the new SN and OSF reconstructions with the reconstructions by Usoskin et al. (2021). The two OSF reconstructions show reasonable agreement in their temporal evolution and statistical characteristics, as illustrated in panels c – d. The corresponding SN reconstructions diverge, particularly during periods of very low solar activity. The SN reconstruction of Usoskin et al. (2021), which is based on a nonlinear regression between OSF and SN, permits negative sunspot numbers; these are interpreted by the authors as being statistically consistent with zero within the stated uncertainties. Thus, while the Usoskin et al. (2021) reconstruction is at first suggestive of large SN variability during grand minima, the SN uncertainty bands are symmetric about zero and therefore positive SN values must also be consistent with zero to within uncertainty. Therefore, the two reconstructions are likely in agreement within their uncertainties. The reliability of the two reconstructions during grand minima cannot be precisely determined, as the directly observed sunspot number record during the Maunder minimum itself is sparse (Muñoz-Jaramillo and Vaquero 2019; Carrasco et al. 2022). Despite these differences, both approaches indicate the persistence of solar magnetic activity during grand minima (Beer, Tobias, and Weiss 1998), as evidenced by the nonzero, finite modulation in open solar flux (panel c).

**Figure 11:**
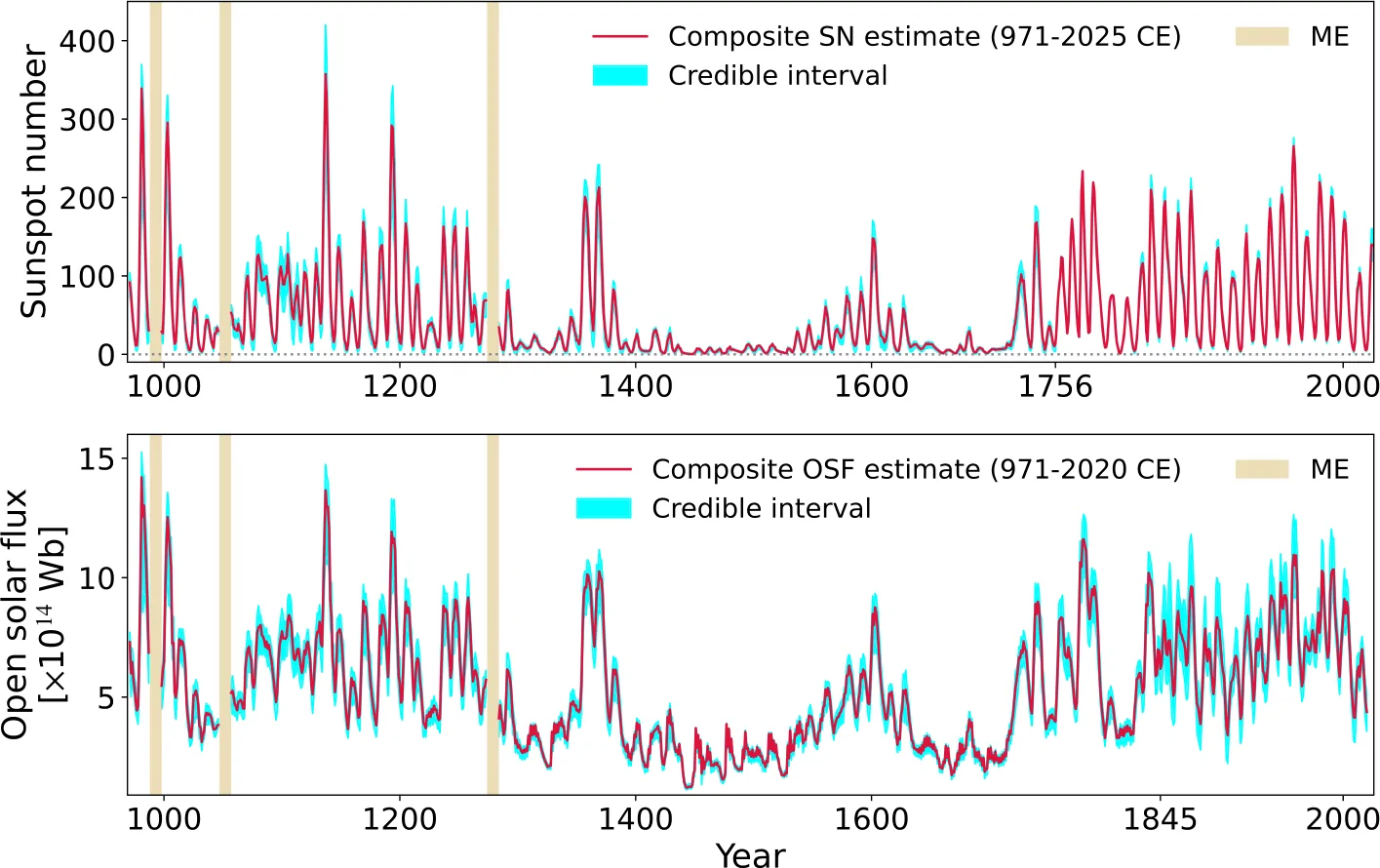
A continuous estimate of the annual SN (top panel) and OSF (bottom panel) since 971 CE, along with associated uncertainties. Top: the segment spanning 971 – 1756 CE is derived from $\phi ^{*}_{B2021}$, while the rest, up to 2025 CE, is the direct SN record maintained by SILSOv2. Bottom: the segment spanning 971 – 1844 CE is derived from $\phi ^{*}_{B2021}$, while the rest is geomagnetic record of OSF from Owens et al. (2024a). Yellow-shaded intervals in both panels denote proposed/confirmed Miyake events during which the reconstructions are masked out.

A continuous estimate of annual SN, together with associated uncertainties, is presented for the period 971 – 2025 CE in Figure 11. This composite record combines our ϕ-based SN reconstruction with the direct sunspot number record from SILSOv2 (Clette and Lefèvre 2015). The ϕ-based reconstruction is truncated at 1756 CE, where it shows good agreement with the overlapping SILSO record (see Figure 9, panel g), and the composite is completed by concatenating the 13-month smoothed yearly SN series from SILSO, which continues until 2025 CE. Three intervals in the vicinity of the possible Miyake events in 993, 1052, and 1279 CE have been excluded from the record, as the uncorrected ϕ during these epochs renders the local reconstruction unreliable.

The results confirm the conclusion of Lockwood, Rouillard, and Finch (2009) that between 1900 and 2020 CE the Sun went through a double-peaked Grand solar maximum. Figure 9 and 11 show other such grand maxima between the Oort and Wolf grand minima and before the Oort minimum. There are somewhat smaller maxima between the Wolf and Spörer minima and between the Maunder and Dalton minima. There is a considerably weaker maximum between the Spörer and Maunder minimum. The peak annual OSF in the 20th century is $10.96^{+1.67}_{-1.46}{\times}10^{14}$ Wb in 1958 CE, which is the largest value since 1200 CE. Before then there are just three years with larger values, the largest being $14.21^{+1.04}_{-4.34}{\times}10^{14}$ Wb in 981 CE. Thus since the start of our data in 971 CE, OSF has ranged between $14.21^{+1.04}_{-4.34}{\times}10^{14}$ Wb and $1.21^{+0.09}_{-0.10}{\times}10^{14}$ Wb and since the end of the Maunder minimum and the present day it has varied between $2.63^{+0.16}_{-0.23}{\times}10^{14}$ Wb and $10.96^{+1.67}_{-1.46}{\times}10^{14}$ Wb. Thus, although we have seen a large fraction of the total range of variation on OSF since 971 CE during the interval for which we have had telescopic observations of sunspots, there were deeper minimum values and higher maximum values before then.

## Discussion and Conclusions

We have demonstrated a new physics-informed inversion technique for reconstructing annually resolved sunspot numbers from heliospheric modulation potential, using ABC. The heliospheric modulation potential can be estimated over the past multiple millennia from cosmogenic isotope proxies, such as the annual relative abundance of ^14^C. The inversion technique consists of two sequential forward models. The first model uses sunspot numbers to compute open solar flux, which is then forward-modelled by the second model to estimate the heliospheric modulation potential. Instead of empirically inverting these causal forward models via regression, we employ a Monte Carlo stochastic forward search to jointly infer probabilistic estimates of sunspot numbers and open solar flux. The forward search begins within a stochastically generated ensemble of sunspot cycle realisations constrained to the space of physically admissible solar histories, propagates the causal chain of forward models, and is followed by ABC rejection sampling with adaptive tolerance defined via rank-based selection. The ensemble of admissible solutions underscores the fundamental non-uniqueness of this inversion and demonstrates that distinct sunspot-number histories can produce similar levels of heliospheric modulation potential.

Several sources of uncertainty should be considered when interpreting the reconstructed record. The forward models linking sunspot number to open solar flux and heliospheric conditions to modulation potential rely on semi-empirical parameterisations that are best constrained by modern ground and space-based observations. We adopt a broad parameter range to avoid over-restrictive priors by taking into account the possibility that solar behaviour in the distant past could differ from the observed telescopic record. The inferred uncertainty intervals primarily reflect stochastic variability among accepted cycle realisations and propagated uncertainty in the target modulation potential. They do not fully incorporate the structural uncertainty associated with alternative model parameterisations. Additional uncertainties arise from the radiocarbon production record itself, including carbon-cycle effects and geomagnetic-field reconstructions. Therefore, the reconstructions should be viewed not as a unique deterministic history but as a probabilistic ensemble of physically consistent solutions.

The reconstructed SN record over the past millennium captures several extended intervals of suppressed solar activity consistent with previously identified grand minima, including the Maunder (17th century) and Spörer (15th century) minima. Crucially, the reconstructed open solar flux remains nonzero throughout these intervals (Figure 9, panel c), corroborating independent evidence for the persistence of weak magnetic cycling during grand minima (Beer, Tobias, and Weiss 1998; Miyahara et al. 2004). This is physically significant – it implies that the solar dynamo does not fully quench during grand minima but instead operates in a reduced but finite activity state, consistent with flux transport dynamo models in which meridional circulation sustains weak cyclic behaviour even at low activity levels and aids in a recovery from grand minima (Saha, Chandra, and Nandy 2022, 2025). The reconstructed cycle amplitudes and durations during these intervals therefore provide empirical constraints on the parameter space of such models – in particular on the minimum OSF consistent with observed cosmogenic isotope modulation. We note, however, that these inferences are subject to an important systematic caveat. The forward model templates for OSF loss rate and HCS tilt are derived exclusively from Solar Cycles 13 – 24, a period of regular solar activity. If grand minimum cycles differ systematically in morphology – for instance, exhibiting lower loss rates or reduced HCS tilt variability – the present framework would not capture this, and the reconstructed cycle amplitudes during such intervals should be interpreted as indicative rather than precise. Addressing this limitation would require independent constraints on cycle morphology specifically during low-activity epochs.

One limitation of the existing regression-based empirical inversions is that they yield unphysical negative sunspot numbers. For use with SN-based total and spectral solar irradiance reconstructions (Vieira et al. 2011; Wu et al. 2018a; Temaj et al. 2026), all SN values below zero have to be addressed (Lockwood and Ball 2020). If they are simply set to zero without also changing the positive values, this will increase the average SN over a solar cycle and thus amplify the associated total solar irradiance (TSI) estimate. The current inversion method eliminates this long-standing issue, and the resulting sunspot numbers remain strictly non-negative throughout the full record. The non-negativity of SN arises naturally from the imposed priors on cycle amplitude, avoiding the need for any post hoc corrections. The imposition of these assumptions does corroborate well with the current best estimate of both ^14^C and sunspots during the Maunder minimum (Carrasco et al. 2022) as will be further investigated in a future study. The resulting reconstruction shows excellent agreement with direct telescopic observations, both at the level of individual solar cycles and beyond. Over the past millennium, the current reconstruction reproduces extended intervals of low and high activity, consistent with previously identified grand minima and grand maxima (Usoskin 2023).

In summary, we have presented a new inversion approach which can reconstruct a physically consistent probabilistic estimation of millennial-scale, annually resolved sunspot numbers from heliospheric modulation potential with an explicit representation of uncertainty. This inference is contingent on the adopted forward models and prior assumptions, and it provides an improved alternative to existing reconstruction methods. Future developments could include applying this inversion technique to longer and older records of cosmogenic isotope-based solar activity proxies.

## Supplementary Information

Below are the links to the electronic supplementary material.

(CSV 147 kB)

(CSV 26 kB)

## Data Availability

Reconstructed sunspot number and open solar flux datasets are provided as supplementary information files. Directly observed sunspot number record used in this work is maintained by the Royal Observatory of Belgium SILSO and is available at www.sidc.be/SILSO/DATA/SN_m_tot_V2.0.csv.

## Materials Availability

NA

## Code Availability

The code for analysis and preliminary visualisation is packaged with all required data here: https://github.com/University-of-Reading-Space-Science/PRISMv1. The Github repository contains the reconstructed SN, OSF, and ϕ estimates.
